# DSS-induced colitis produces inflammation-induced bone loss while irisin treatment mitigates the inflammatory state in both gut and bone

**DOI:** 10.1038/s41598-019-51550-w

**Published:** 2019-10-22

**Authors:** Corinne E. Metzger, S. Anand Narayanan, Jon P. Elizondo, Anne Michal Carter, David C. Zawieja, Harry A. Hogan, Susan A. Bloomfield

**Affiliations:** 1 Department of Health and Kinesiology, Texas A&M University, College Station, TX USA; 2 Department of Medical Physiology, Texas A&M University - Health Science Center, Temple, TX USA; 3 Departments of Mechanical/Biomedical Engineering, Texas A&M University, College Station, TX, USA

**Keywords:** Ulcerative colitis, Osteoimmunology

## Abstract

Chronic pediatric inflammatory bowel disease (IBD) leads to lack of bone accrual, bone loss, and increased fractures. Presently there is no cure, and many IBD treatments incur negative side effects. We previously discovered treatment with exogenous irisin resolved inflammatory changes in the colon, gut lymphatics, and bone in a mild IBD rodent model. Here we assess irisin treatment in severe IBD induced via dextran sodium sulfate (DSS). Male Sprague Dawley rats (2-mo-old) were untreated (Con) or given 2% DSS in drinking water. In week two, half of each group (Con + Ir and DSS + Ir) received injections of recombinant irisin (i.p., 2x/wk). After 4 weeks, gut inflammation was associated with declines in bone mineral density and cancellous bone volume. Furthermore, elevated osteocyte TNF-α, interleukin-6, RANKL, OPG, and sclerostin corresponded with higher osteoclast surfaces and lower bone formation rate in DSS animals as well as lower ultimate load. While irisin treatment improved colon inflammation, there were no improvements in bone density or bone mechanical properties; however, irisin elevated bone formation rate, decreased osteoclast surfaces, and reduced osteocyte pro-inflammatory factors. These data highlight the negative impact of chronic gut inflammation on bone as well as the therapeutic potential of irisin as an anti-inflammatory treatment.

## Introduction

Inflammatory bowel disease (IBD), consisting of Crohn’s disease and ulcerative colitis, is increasing in incidence worldwide^1–4^. Particularly concerning is the increasing incidence of pediatric-onset IBD, which is typically characterized by a more extensive phenotype and life-long complications^5^. In the United States, an estimated 1.6 million individuals have IBD, with approximately 80,000 of those being children/adolescents^6^. While the exact etiology of IBD remains largely unknown, it is believed to be in part an autoimmune reaction against tissues within the gastrointestinal tract leading to chronic inflammation and extra-intestinal comorbidities, such as inflammation-induced bone loss^7,8^.

A high percentage of patients with IBD present with osteopenia or osteoporosis^9–14^, with this bone loss independent of corticosteroid use^10,11,15^. Additionally, children and adolescents with IBD have lower bone mass than age-matched healthy controls^16–19^ with this decrement in bone mass persisting into adulthood^20,21^. The low bone mass in IBD for all patient populations correlates with a 40% higher incidence of fractures than the general population^22^. A large cohort study determined the risk for hip fractures in patients with IBD is 60% higher compared to the risk in healthy populations^23^ and vertebral fractures are present in approximately 20% of patients with IBD, even in those under the age of 30^24^.

Animal models of IBD can recapitulate loss of bone and alterations in bone turnover due to gut inflammation^25–27^. Two chemical methods to induce gut inflammation in animal models include induction via 2,4,6-Trinitrobenzenesulfonic acid (TNBS) and dextran sodium sulfate (DSS) treatment. TNBS is believed to cause a T-cell mediated response against hapten-modified proteins on the gut lumen, while DSS is hypothesized to be directly toxic to epithelial cells specific to the colon^28,29^. The TNBS-induction method is considered to model human Crohn’s, while DSS models human ulcerative colitis^30^. Previously, we demonstrated for the first time in the TNBS model that immune adaptations in the gut coincide with a lymphatic architecture phenotype analogous to what is seen in human Crohn’s disease^31^. Additionally, we found these same young TNBS rats exhibited increased osteocyte protein expression of pro-inflammatory cytokines, high osteoclast surfaces, and low bone formation rate^31^. However, the TNBS dosing we utilized did not result in significant loss of bone mass and, therefore, did not effectively model the declines in bone mechanical properties as seen in human clinical populations. Other rodent models for IBD have demonstrated bone loss with chronic inflammation from experimentally-induced colitis and overexpression of pro-inflammatory mediators like interleukin-6^25,27,32^ however, the dynamics of how immune dysregulation in the context of IBD influences the degree of bone loss is still not fully understood.

Furthermore, as a chronic disease with no cure at this time, treatments to mitigate inflammation and disease symptoms in IBD are greatly needed. Many current treatments have negative side effects, including exacerbated bone loss with corticosteroids^33,34^ and increased risks of serious infections and malignancies with anti-TNF therapy^35–38^. Previously, we examined whether exogenous treatment with irisin, a myokine released from exercising muscle, could alleviate the inflammatory insult in the gut and bone^39^. We found reductions in gut and osteocyte pro-inflammatory cytokines, as well as increased bone formation and decreased osteoclast surfaces in irisin-treated TNBS rats^39^. While the direct roles of irisin in bone remain to be fully elucidated^40–43^, our work along with others have demonstrated the its potential to modulate inflammation^44–46^.

The goals of this current project were two-fold: first, we aimed to examine the impact of chronic ulcerative colitis, utilizing dextran sodium sulfate (DSS) to induce gut inflammation, on bone cellular and mechanical properties. Secondly, our goal was to determine the impact of treatment with exogenous irisin on bone outcomes in young rats. We hypothesized that DSS animals, when compared to age-matched controls, would exhibit lower bone formation rate and higher osteoclast surfaces and that DSS would lead to gut lymphatic hyperproliferation and pro-inflammatory phenotype. In addition, we expected higher osteocyte pro-inflammatory cytokine protein expression (tumor necrosis factor-α and interleukin-6) as well as elevations in osteocyte protein regulators of bone turnover (RANKL, OPG, and sclerostin) and elevated osteocyte apoptosis (measured via annexin V). We expected this would result in significantly lower bone mineral density and cancellous bone volume, with corresponding declines in bone mechanical properties. Secondly, we hypothesized that treatment with irisin would ameliorate the gut and bone pathological adaptations and mitigate declines in bone mass and mechanical properties.

## Results

### Animals

There were no differences among animal groups in body weight or food intake at baseline. Beginning during the second week of DSS, all DSS animals had lower body weight than controls and remained at a lower body weight for the rest of the study (Supplemental Table 1]. Con animals gained approximately 60% of their initial body weight while DSS animal only gained 40%. A decline in food intake also began in the second week of DSS treatment, approximately 20% lower food intake; however, by the final week food intake was not different between the four groups (Supplemental Table 2]. Also beginning in the second week of treatment, many DSS animals experienced bloody stools and diarrhea that lasted throughout the remainder of the protocol.

### Histopathology of the colon is elevated in DSS animals

**Figure 1:**
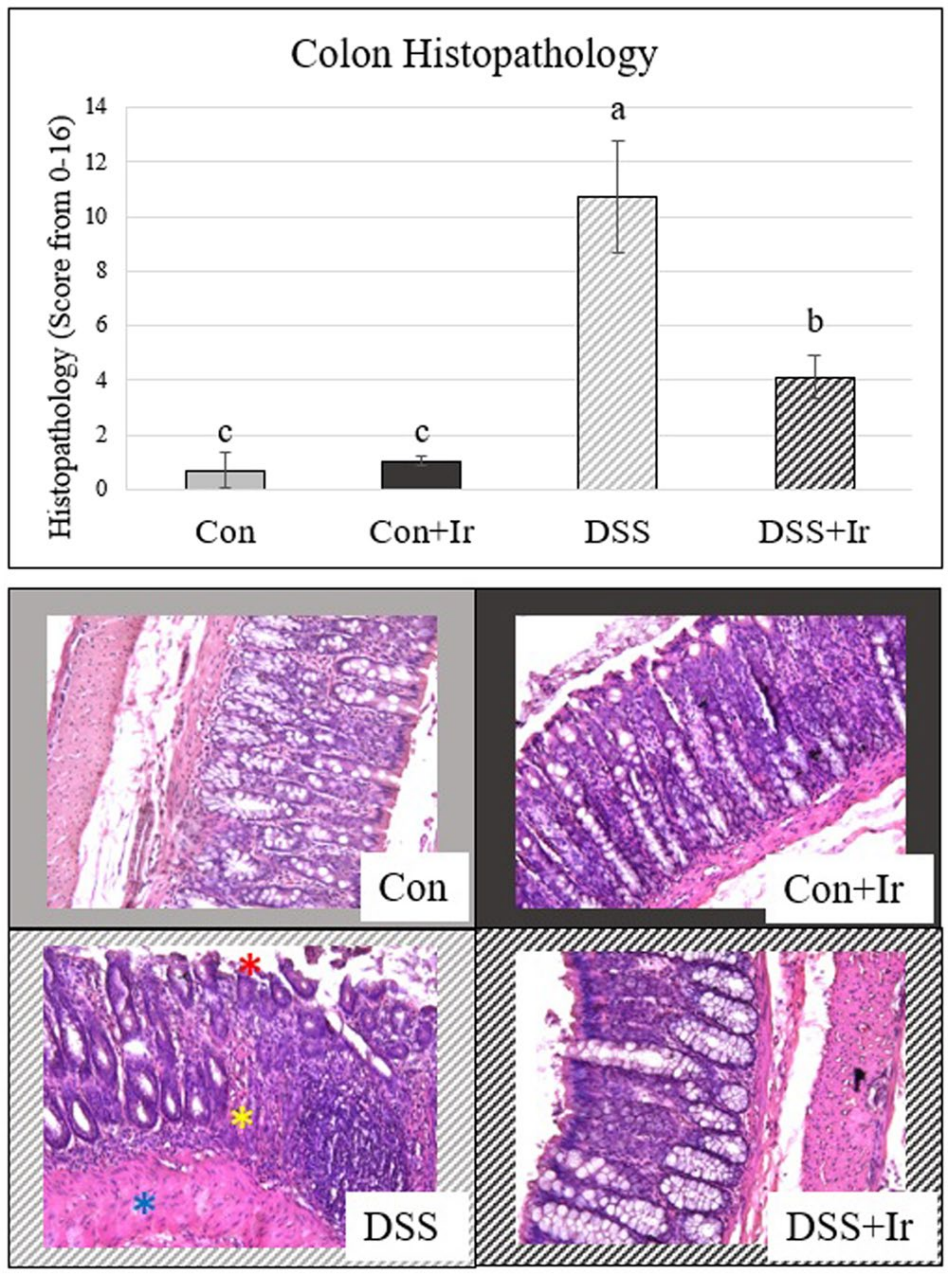
Colon histopathology (n = 8/group). (**A**) Colon aggregated histopathology scores. (**B**) Representative images of colon histopathology. The DSS image is highlighted by features assessed and scored: red asterisks reflects fragmentation and loss of the intestinal epithelial layer, yellow asterisks reflects loss of crypts of Lieberkuhn and breach of immune cells into the mucosal compartment (i.e. increased cellularity), and the blue asterisks highlight the muscularis externa and observed thickening. Values are presented as mean ± SD. Bars not sharing the same letter are statistically different (p < 0.05).

Two-way ANOVA of colon histopathology scores revealed main effects of DSS, irisin, and a DSS*irisin interaction effect (p < 0.0001 for all) (Fig. 1). DSS animals had a histopathology score of over 10, statistically elevated compared with Con, Con + Ir, and DSS + Ir groups. Irisin-treated DSS animals (DSS + Ir) had histopathology scores greater than both Con groups, but much lower than those for untreated DSS animals.

### DSS colitis resulted in modest infiltration of podoplanin^±^ structures into the mucosal compartment, with no changes in lymphatic architecture in the submucosal compartment

**Figure 2:**
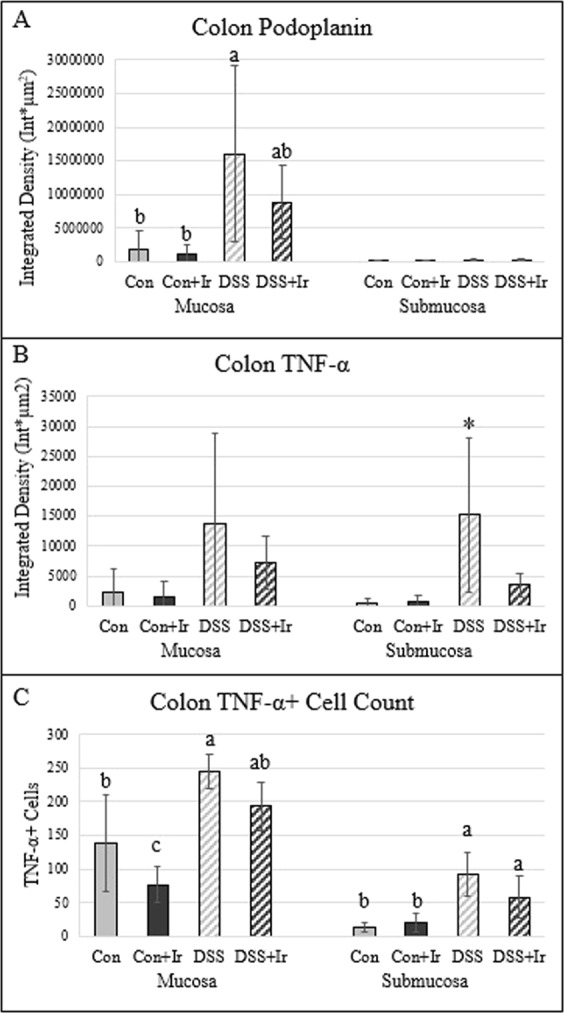
Colon lymphatic architecture and TNF-α expression (n = 5/group). (**A**) Colonic mucosa and submucosa podoplanin expression, a lymphatic endothelial marker, integrated density (expression x area). (**B**) Colonic mucosa and submucosa TNF-α integrated density, (**C**) absolute number of TNF-α + cells in the colonic compartments. Values are presented as mean ± SD. *Indicates statistically different from all other groups (p < 0.05). Bars not sharing the same letter are statistically different (p < 0.05). See Supplemental Data for representative images.

In the colon mucosa, integrated density for podoplanin, a canonical lymphatic endothelial marker, had main effects for DSS (p = 0.007) with no effect of irisin (p = 0.281) nor an interaction effect (p = 0.365) (Fig. 2A). The DSS group average was higher than all other groups. In the submucosa, there were no differences among groups (ANOVA p = 0.124).

### The increased DSS podoplanin^±^ density is associated with elevated TNF-α

In the colon mucosa, there were not statistical differences in integrated density for TNF-α (p = 0.130) (Fig. 2B); there were main effects of DSS (p < 0.0001) and irisin (p = 0.014) on TNF-α + cell count, but no interaction effect (p = 0.809). For mucosa TNF-α^+^ cell count, DSS group values were higher than those for both Con groups (Fig. 2C]. For the submucosa integrated density for TNF-α, there was a main effect of DSS effect (p = 0.009) with no effect of irisin (p = 0.063) or interaction (p = 0.059) (Fig. 2B]. The DSS group TNF-α integrated density in the submucosa was higher than all other groups (Fig. 2B]. For submucosa TNF-α^+^ cell count, there was a main effect of DSS (p < 0.0001) with no effect of irisin (p = 0.265) and no interaction (p = 0.087) with both DSS groups’ values higher than those for both Con groups (Fig. 2C].

### DSS resulted in declines in volumetric bone mineral density at both the proximal tibia metaphysis and femoral neck

Total bone mineral content (BMC) at the proximal tibia metaphysis was lower in both DSS groups compared to Con with Con + Ir not different from either group (Table 1]. There was a main effect of DSS (p = 0.009), but no effect of irisin (p = 0.179) or an interaction (p = 0.646). Similar effects were seen with proximal tibia total volumetric bone mineral density (vBMD) with a main effect of DSS (p = 0.003) and no effect of irisin (p = 0.914) and no interaction effect (p = 0.723) (Table 1]. Both Con groups had higher total vBMD than both DSS groups. Cancellous vBMD was not different among the four groups (ANOVA p = 0.123). There was a main effect of DSS on metaphyseal cortical vBMD (p = 0.004) (Table 1], but no effects of irisin (p = 0.711) nor an interaction (p = 0.468). Metaphyseal cortical vBMD was lower in DSS + Ir than in both Con groups, while untreated DSS values were not different from any group (Table 1]. At the femoral neck, there was a main effect of DSS on total BMC (p = 0.001) and no irisin or interaction effects (p = 0.537, p = 0.632, respectively). Both DSS groups’ values for total BMC were lower than those for both Con groups. In total vBMD at the femoral neck, there was also a main effect of DSS (p = 0.003) and no irisin or interaction effects (p = 0.624, p = 0.317, respectively). Both Con groups’ values were higher than those for the untreated DSS animals, with DSS + Ir values not different from those for any other group. There was also a main effect of DSS on femoral neck cancellous vBMD (p < 0.0001) with both DSS groups lower than both Con groups but no main effect of irisin (p = 0.940) and no interaction effect (p = 0.493). Metaphyseal cortical vBMD at the femoral neck was not statistically different among groups (p = 0.149; Table 1).

**Table 1:** Peripheral quantitative computed tomography of the proximal tibia metaphysis and femoral neck.

Group	Total BMC (g)	Total vBMD (mg/cm^3^)	Cancellous vBMD (mg/cm^3^)	Metaphyseal Cortical vBMD (mg/cm^3^)
***Proximal Tibia Metaphysis***				
Con	7.99 ± 1.05^a^	444 ± 25^a^	187 ± 60	462 ± 24^a^
Con + Ir	7.46 ± 0.61^ab^	447 ± 21^a^	150 ± 45	466 ± 21^a^
DSS	7.04 ± 0.51^b^	413 ± 28^b^	144 ± 25	437 ± 26^ab^
DSS + Ir	6.77 ± 0.63^b^	408 ± 35^b^	133 ± 27	425 ± 36^b^
***Femoral Neck***				
Con	3.91 ± 0.38^a^	956 ± 35^a^	787 ± 33^a^	1220 ± 23
Con + Ir	3.89 ± 0.53^a^	942 ± 44^a^	773 ± 44^a^	1221 ± 39
DSS	3.45 ± 0.28^b^	846 ± 101^a^	680 ± 62^b^	1171 ± 83
DSS + Ir	3.33 ± 0.17^b^	884 ± 78^ab^	698 ± 84^b^	1186 ± 35

Groups not sharing the same letter are statistically different.

### Midshaft tibia cortical bone mineral content and area were lower in all treatment groups compared to control

At the cortical midshaft for total BMC, there was a main effect of DSS (p = 0.025) with no irisin effect (p = 0.369) nor interaction effect (p = 0.055) (see Table 2]. The Con group mean was higher than those for all other groups. Midshaft cortical vBMD was not statistically different among the 4 groups (p = 0.079). Total area was also higher in Con animals compared to that in all other groups, with a main effect of DSS (p = 0.011) and no irisin or interaction effects (p = 0.095, p = 0.171, respectively; Table 2).

**Table 2:** Midshaft tibia pQCT and mechanical properties.

Group	Cortical BMC (g)	Cortical vBMD (mg/cm^3^)	Total Area	Stiffness (N/mm)	Elastic Modulus (GPa)	Ultimate Stress (MPa)
Con	6.3 ± 0.3*	1367 ± 11	6.2 ± 0.4*	213.2 ± 23.8	8.5 ± 1.0^b^	170.5 ± 17.0
Con + Ir	6.0 ± 0.2	1370 ± 12	5.8 ± 0.4	196.9 ± 25.4	9.1 ± 0.8^ab^	177.0 ± 11.4
DSS	5.8 ± 0.3	1369 ± 11	5.6 ± 0.3	209.1 ± 19.8	10.0 ± 1.5^a^	181.0 ± 27.2
DSS + Ir	6.0 ± 0.3	1383 ± 9	5.6 ± 0.3	205.6 ± 16.6	10.0 ± 0.7^a^	187.8 ± 11.9

*Different from all other groups. Groups not sharing the same letter are statistically different.

### Ultimate load was decreased in DSS at both the midshaft tibia and femoral neck

**Figure 3:**
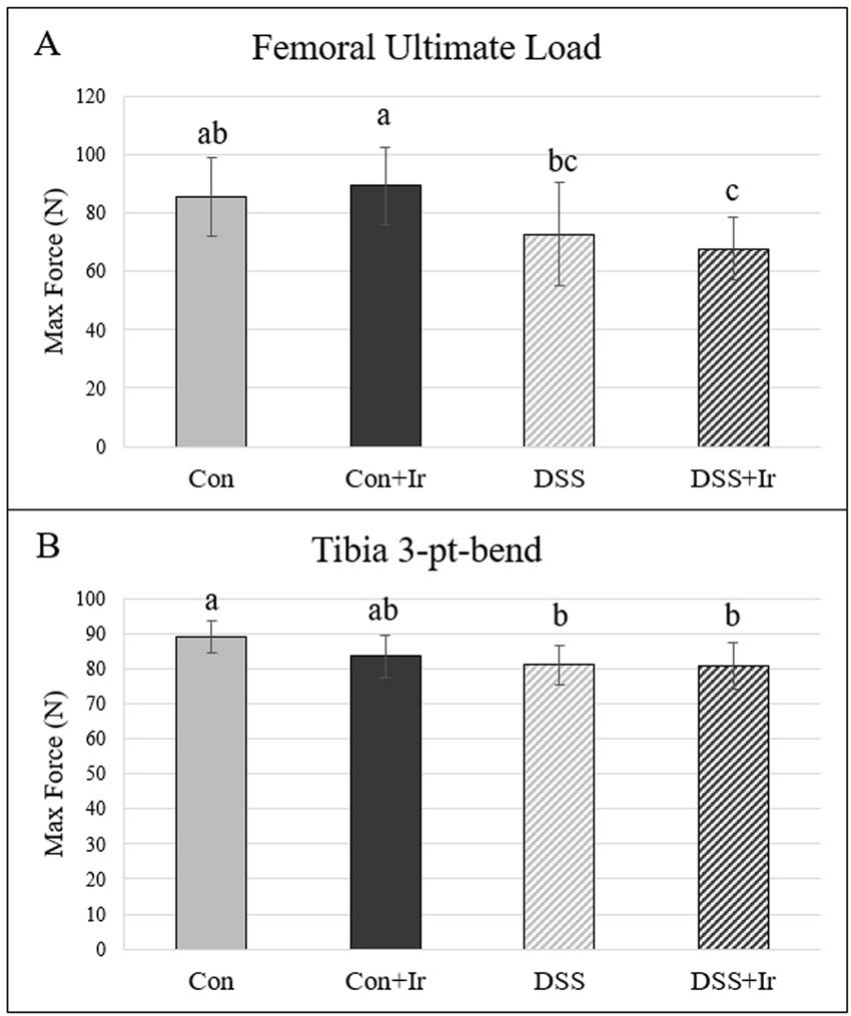
Mechanical testing for estimated ultimate load (N) at the femoral neck and midshaft tibia via three-point-bend (n = 8/group). (**A**) At the femoral neck, ultimate load was lower in both DSS groups compared to Con + Ir with Con not different than DSS and DSS + Ir not different than DSS alone. (**B**) Both DSS and DSS + Ir groups showed decreased ultimate load from the 3-pt bending test compared to Con. Data are presented as mean ± SD. Bars not sharing the same letter are statistically different (p < 0.05).

At the femoral neck, there was a main effect of DSS on ultimate load (p = 0.004) with no effect of irisin (p = 0.928) and no interaction effect (p = 0.413) (Fig. 3A]. The Con + Ir group mean was higher than both DSS groups with Con higher than DSS + Ir, but not statistically different from DSS. At the midshaft tibia, there was also a main effect of DSS on ultimate load (p = 0.02) with no irisin effect (p = 0.201) and no interaction effect (p = 0.232). The Con group mean was higher than that of both DSS groups, with the Con + Ir mean not different from those of any group (Fig. 3B]. At the midshaft tibia, there was also a main effect of DSS on modulus (p = 0.003), but no effect of irisin (p = 0.519) or an interaction (p = 0.450) (Table 2]. The Con group mean was lower than that of both DSS groups, with the Con + Ir mean not different from any group. At the midshaft tibia, there were no differences in stiffness (ANOVA p = 0.485) or in ultimate stress (p = 0.359; Table 2).

### DSS resulted in significantly lower cancellous BV/TV at the proximal tibia, L4, and femoral neck, but irisin treatment mitigated declines in osteoid surface and restored osteoclast surfaces to control levels

**Figure 4:**
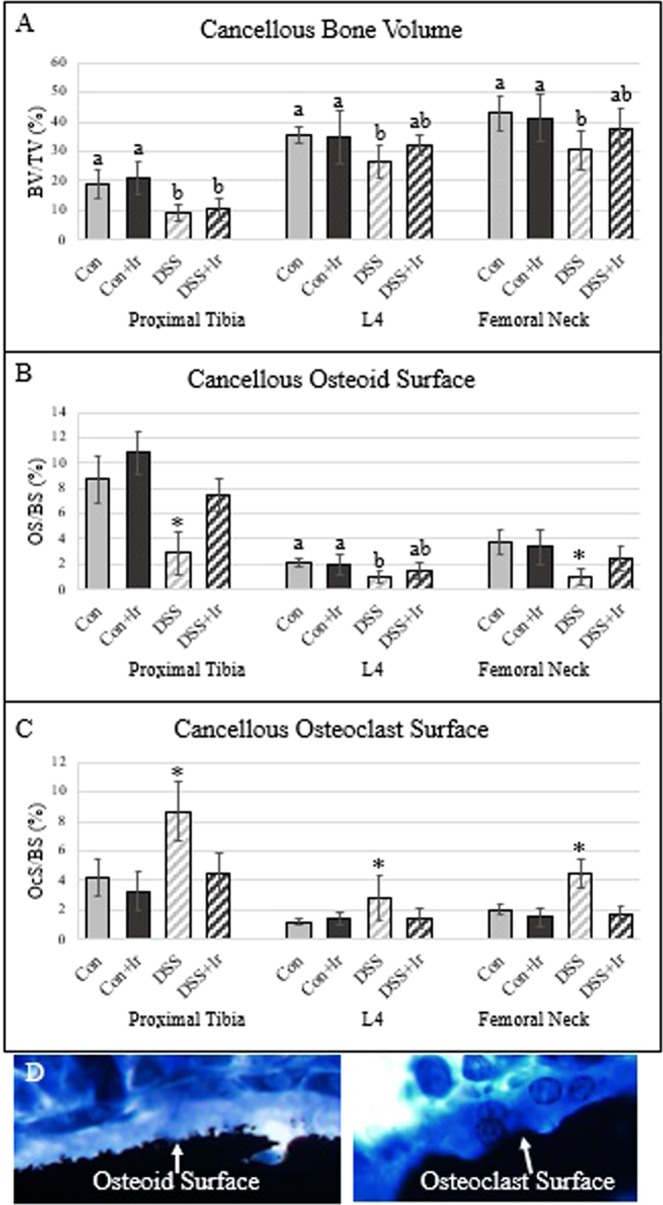
Static cancellous histomorphometry of the proximal tibia, 4^th^ lumbar vertebrae, and femoral neck (n = 8/group). (**A**) Cancellous bone volume was lower in DSS compared with Con and Con + Ir in all three bone compartments, with irisin treatment in the DSS group not different from Con groups or DSS in L4 and femoral neck. (**B**) Cancellous osteoid surface was lower in DSS compared to Con groups in all three bone sites, with osteoid surface higher than DSS and not different from Con in DSS + Ir at the proximal tibia and femoral neck. (**C**) Cancellous osteoclast surface was elevated in DSS compared to all other groups at all three bone sites, with the DSS + Ir group not different than Con in all three bone sites. (**D**) Representative image of osteoid surface and osteoclast surface (bone is black, white arrow indicates osteoid/osteoclast surface). Data are presented as mean ± SD. *Indicates statistically different from all other groups within the bone site (p < 0.05). Bars not sharing the same letter are statistically different within in the bone site (p < 0.05).

There were main effects for DSS on cancellous BV/TV at the proximal tibia (p < 0.0001), L4 (p = 0.008), and the femoral neck (p = 0.015) (Fig. 4A]. At the proximal tibia, both Con groups had higher BV/TV than both DSS groups. In both the 4^th^ lumbar vertebra and the femoral neck, both Con groups exhibited higher BV/TV values than did DSS animals, with the DSS + Ir mean not different from those of any group. For osteoid surface, at the proximal tibia there were main effects of both DSS (p < 0.0001) and irisin (p = 0.005) and an interaction effect (p < 0.0001), with untreated DSS values lower than those of all other groups (Fig. 4B]. At L4, there was a main effect of DSS (p = 0.001) but no effect of irisin (p = 0.275) nor an interaction effect (p = 0.132). At L4, both Con groups had higher mean % osteoid surface than did DSS animals, with the DSS + Ir mean not different from those of any group. At the femoral neck, there was a main effect of DSS (p < 0.0001), no effect of irisin (p = 0.219), and an interaction effect (p = 0.049) where DSS values were lower than those for all other groups. For osteoclast surface at the proximal tibia, there were significant main effects for DSS and irisin (p < 0.0001 for both) and an interaction effect (p = 0.005) (Fig. 4C]. At L4 for osteoclast surface, there were main effects of DSS (p = 0.014), no effect of irisin (p = 0.065), and a significant interaction effect (p = 0.015). At the femoral neck, there were significant main effects of DSS and irisin and an interaction effect (p < 0.0001). At all bone sites, untreated DSS animals exhibited higher % osteoclast surface compared to all other groups.

### DSS resulted in lower cancellous bone formation rate while irisin treatment resulted in higher bone formation rate at two of the three bone sites

**Figure 5:**
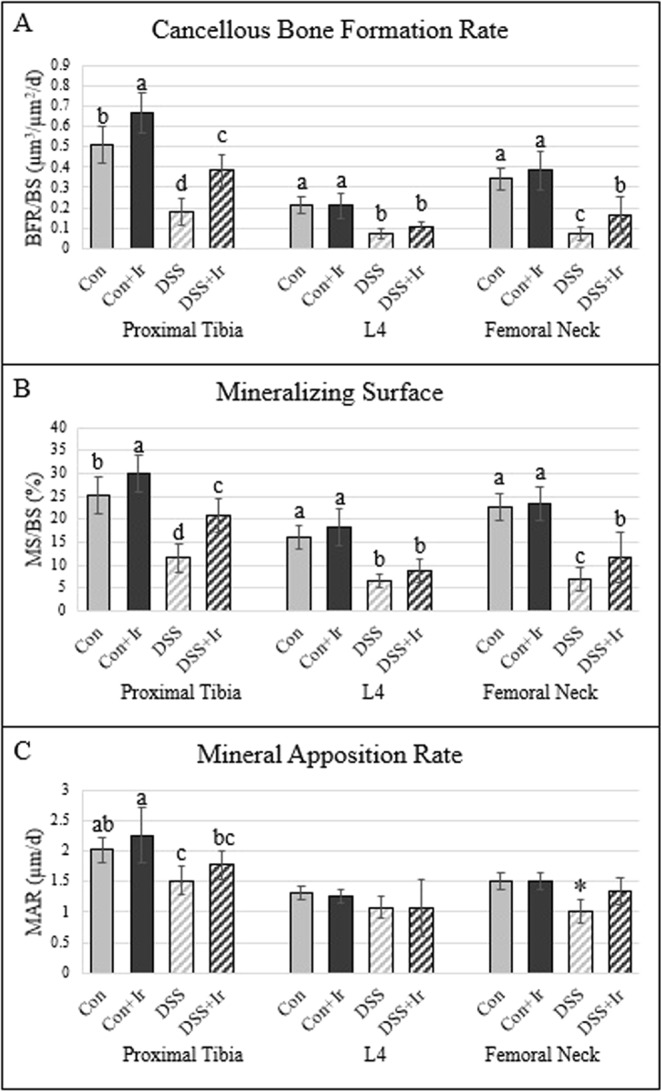
Dynamic cancellous histomorphometry of the proximal tibia, 4th lumbar vertebrae, and femoral neck (n = 8/group). (**A**) Cancellous bone formation rate was lower in DSS compared to both Con groups in all three bone compartments, with irisin treatment in the DSS group showing modest improvements in BFR at the proximal tibia and femoral neck. (**B**) Mineralizing surface was lower in DSS compared to both Con groups in all three bone sites, with DSS + Ir demonstrating increased MS/BS compared to DSS alone at the proximal tibia and femoral neck. (**C**) Mineral apposition rate was decreased in DSS compared to Con groups in the proximal tibia and femoral neck, with a modest increase in MAR in the DSS + Ir group at the proximal tibia and not different from Control groups in the femoral neck. There were not differences at L4. Data are presented as mean ± SD. *Indicates statistically different from all other groups within the bone site (p < 0.05). Bars not sharing the same letter are statistically different within the bone site (p < 0.05).

At the proximal tibia, there were main effects of BFR/BS for both DSS and irisin (p < 0.0001 for both), but no interaction effect (p = 0.588) (Fig. 5A]. The Con + Ir animals exhibited the highest BFR/BS, followed by Con, DSS + Ir, and DSS groups, with all group means statistically different from each other. There were main effects for DSS in both MS/BS and MAR (p < 0.0001 for both) and main effects of irisin for MS/BS and MAR (p < 0.0001, p = 0.027, respectively). There were no DSS*Irisin interaction effects for proximal tibia MS/BS or MAR (p = 0.097, p = 0.911, respectively). For MS/BS, DSS was lower than Con and irisin treatment increased MS/BS compared to the respective untreated groups, both in DSS and Con (Fig. 5B]. For MAR, DSS was lower than Con with slight increases due to irisin treatment in both Con + Ir and DSS + Ir compared to the respective untreated group (Fig. 5C]. At L4, BFR/BS was higher in both Con groups compared to both DSS groups (Fig. 5A]. There was a main effect of only DSS at this site on BFR/BS (p < 0.0001), with no effects of irisin (p = 0.271) nor an interaction effect (p = 0.234). For MS/BS, there was a main effect of DSS (p < 0.0001) and no effects of irisin (p = 0.058) and no interaction (p = 0.964). Both DSS groups were lower than both Con groups (Fig. 5B]. There were no statistical differences in L4 MAR (ANOVA p = 0.117; Fig. 5C). In the femoral neck cancellous bone, there was a main effect of DSS (p < 0.0001) and irisin (p = 0.044) and no interaction effect (p = 0.435). Both Con groups had higher BFR/BS than did DSS + Ir animals, whose group mean was in turn higher than that for DSS alone (Fig. 5A]. For femoral neck MS/BS, there was a main effect of DSS (p < 0.0001) and no effect of irisin (p = 0.098) or an interaction (p = 0.203). Both DSS groups had lower MS/BS compared to Con while DSS + Ir was higher than DSS alone (Fig. 5B]. For femoral neck MAR, there were main effects of DSS (p < 0.0001) and irisin (p = 0.024) and a significant DSS*Irisin interaction effect (p = 0.023). DSS values for MAR were lower than those for all other groups at this site (Fig. 5C].

### At the tibia midshaft, periosteal bone formation rate was lower in DSS animals, with modest improvements in mineral apposition rate in irisin-treated DSS rats

**Figure 6:**
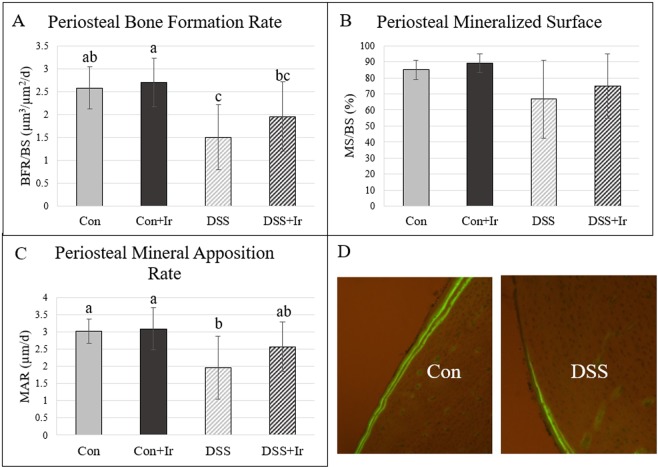
Dynamic cortical histomorphometry of the midshaft tibia (n = 8/group). (**A**) Cortical bone formation rate was lower in DSS compared with Con and Con + Ir, with DSS + Ir not different from Con or DSS alone. (**B**) There were not statistical differences in mineralizing surface between the four groups. (**C**) Mineral apposition rate was decreased in DSS compared to both Con groups while DSS + Ir was not different from Con or DSS alone. (**D**) Representative images of periosteal BFR in Con vs. DSS. Data are presented as mean ± SD. Bars not sharing the same letter are statistically different (p < 0.05).

There was a main effect of DSS (p = 0.001) on mid-shaft tibia periosteal BFR and no effects of irisin (p = 0.239) or an interaction effect (p = 0.501) (Fig. 6A]. DSS animals exhibited the lowest periosteal BFR; irisin treatment in DSS exerted a modest effect, with DSS + Ir values not significantly different from DSS nor from Con group means. MS/BS was not statistically different across groups at the tibia midshaft (p = 0.073; Fig. 6B). There were main effects of DSS on MAR (p = 0.006), but no effects of irisin (p = 0.217) and no DSS*Irisin interaction effect (p = 0.338). The untreated DSS group exhibited lower MAR values than those for both Con groups, with the DSS + Ir mean not different from that of any group (Fig. 6C].

### DSS resulted in higher osteocyte inflammatory markers, RANKL and OPG, and sclerostin. Irisin treatment in DSS mitigated these increases

#### TNF-α

**Figure 7:**
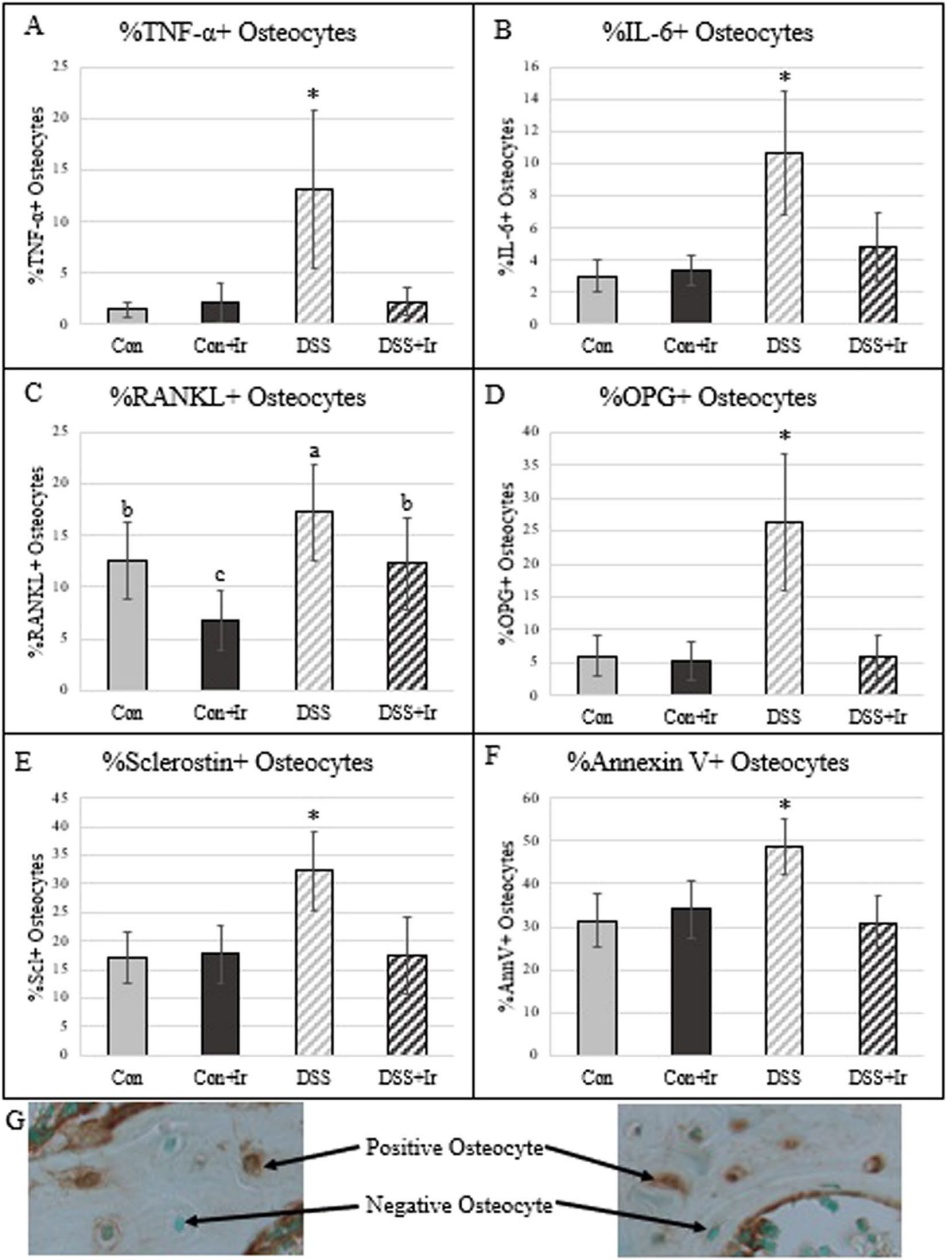
Immunohistochemistry of osteocytes in the cancellous bone of the distal femur (n = 8/group). (**A**) %TNF-α-positive osteocytes were higher in DSS compared to all other groups. B) %IL-6-positive osteocytes were higher in DSS compared to all other groups. (**C**) %RANKL-positive osteocytes were highest in DSS compared to all other groups, while Con + Ir was lower than Con alone and DSS + Ir was not different from Con. (**D**) %OPG-positive osteocytes were higher in DSS compared to all other groups. (**E**) %Sclerostin-positive osteocytes were higher in DSS compared to all other groups. (**F**) %Annexin-V-positive osteocytes were higher in DSS compared to all other groups. (**G**) Representative images of osteocyte immunohistochemistry (image shown represents sclerostin stain). Data are presented as mean ± SD. *Indicates statistically different from all other groups (p < 0.05). Bars not sharing the same letter are statistically different (p < 0.05).

There were main effects of both DSS and irisin-treatment (p = 0.001, p = 0.002, respectively) on %TNF-α + osteocytes as well as an interaction effect (p = 0.001) (Fig. 7A]. The DSS group exhibited higher values than did all other groups, with the DSS + Ir group mean not different from that of the Con group.

#### IL-6

For %IL-6 + osteocytes there was a main effect of DSS treatment (p < 0.0001) and irisin (p = 0.003) as well as an interaction effect (p = 0.001) (Fig. 7B]. The DSS group had the highest % IL-6 + osteocytes mean as compared to all other groups.

#### RANKL

There was a main effect of DSS (p = 0.002) on %RANKL + osteocytes and an effect of irisin (p = 0.001) with no interaction effect (p = 0.776) (Fig. 7C]. DSS animals had the highest %RANKL + osteocytes mean; both irisin-treated group means for %RANKL + osteocytes were lower than those for their respective control groups (Con and DSS).

#### OPG

Main effects for DSS and irisin-treatment were present in %OPG + osteocytes as well as an interaction effect (p < 0.0001 for all) (Fig. 7D]. The DSS group had the highest mean %OPG + osteocytes of all groups; irisin-treated DSS animals exhibited values no different from those of both Con groups.

#### Sclerostin

There were main effects of both DSS (p = 0.002) and irisin (p = 0.003) and a DSS*Irisin interaction effect (p = 0.002) for sclerostin-positive osteocytes (Fig. 7E]. The DSS group had the highest % sclerostin + osteocytes of all groups; irisin-treated DSS animals exhibited values no different from those of both Con groups.

#### Annexin V

There was a main effect of annexin V-positive osteocytes for DSS (p = 0.012) and irisin (p = 0.006) and an interaction effect (p = 0.001) (Fig. 7F]. The DSS group had the highest annexin V positive osteocytes of all groups. DSS + Ir values were not different from either Con group.

#### Both body weight and colon histopathology scores statistically predicted max force

From linear regression models, end-point body weight statistically predicted ultimate load of the mid-shaft tibia (R = 0.422, R^2^ = 0.178, p = 0.028) and the femoral neck (R = 0.561, R^2^ = 0.315, p = 0.002). Gut histopathology scores significantly predicted ultimate load at both the tibia (R = 0.474, R^2^ = 0.225, p = 0.026) and femoral neck (R = 0.436, R^2^ = 0.190, p = 0.043) as well. Multiple linear regression with both body weight and colon histopathology did not meet our requirements for acceptance.

#### Colon histopathology significantly predicted TNF-α-positive osteocytes

Colon histopathology as the independent variable in linear regression statistically predicted approximately 63% of the variability in osteocyte TNF-α (R = 0.797, R^2^ = 0.635, p < 0.0001).

## Discussion

The primary findings of this study are, first, chronic DSS-induced IBD in young rats resulted in impaired gains in body weight, lower vBMD and cancellous bone volume at multiple bone sites, and decreased ultimate load. These changes were matched with high osteoclast surfaces and low bone formation rate, as well as increased osteocyte pro-inflammatory cytokines. Secondly, exogenous treatment with irisin ameliorated inflammatory changes in bone turnover and osteocyte proteins but did not protect against losses in bone mass or mechanical properties. Overall, these data confirm the negative consequences of chronic gut inflammation on bone and highlight the potential for irisin treatment to mitigate inflammatory bone changes.

Although the gut histopathology score was much greater in this model compared with a TNBS-Crohn’s disease rodent model^39^, the degree of colonic lymphatic architecture changes and associated inflammation were more modest with DSS. The role of lymphatics in the context of IBD was identified in the first description of the disease by Crohn in 1932^47^. Lymphatic remodeling has been speculated to be compensatory to the chronic inflammation along the digestive system, with both Crohn’s disease and ulcerative colitis patients exhibiting an increased density of lymphatic vessels. Interestingly, in Crohn’s disease the lymphatic hyperproliferation is seen transmurally along the gut compartments (i.e. in both the mucosa and submucosa), whereas lymphatic remodeling in ulcerative colitis restricted to the mucosa^48^. This compartment-specific architectural remodeling in the gut wall observed in humans with Crohn’s disease and ulcerative colitis is reflected in our previously used TNBS-induced animal model for Crohn’s disease^39^ and with this DSS-induced colitis model. The current study demonstrates lymphatic hyperproliferation primarily in the mucosal compartment. The location of lymphatic remodeling, as detected by elevated podoplanin + expression, may be related to the location of inflammatory response, as we see a stronger presence of TNF-α in the colonic mucosa compared with the submucosa. TNF-α induces formation of new lymphatic vessels in pathological conditions, with mice overexpressing TNF-α displaying abnormal gut lymphatics^49,50^. To our knowledge, this is the first investigation of lymphatic alterations in the gut of DSS-induced colitis, and our results aid in further understanding of DSS as a model of human ulcerative colitis.

Similar to other animal models of IBD, the rats in our study had significantly lower body weight than age-matched controls^25,26^. In our study, both control groups gained an average of 60% of their initial bodyweight over the four-week experimental period while the combined DSS groups gained an average of 37%. Concurrent with the diminished gain of body weight was a reduction in vBMD at both the proximal tibia (−7%) and the femoral neck (−11%) in DSS rats compared to controls. Additionally, cancellous bone volume in the DSS group was 51% lower at the proximal tibia, 25% lower in the 4^th^ lumbar vertebra, and 29% lower in the femoral neck compared to control values. Compared to the healthy age-matched population, pediatric patients with IBD typically exhibit lower body weight, total cross-sectional area of bone^51^, cancellous BMD^17^, and total body bone mass^16,18,19^. Since approximately 35% of total bone mineral accrual occurs during adolescence^52^, IBD during these critical years results in impaired peak bone mass in young adulthood^20,21^. Our DSS rat model, tested at an age relevant to the pediatric patient, exhibits similar findings with chronic gut inflammation leading to impairments in body weight gain and bone mass.

The lower bone mass in the DSS rats was mediated by approximately 2-fold higher cancellous osteoclast-covered surfaces at the proximal tibia, 4^th^ lumbar vertebra, and femoral neck and by 64–79% lower bone formation rate at each site. In patients with IBD, serum markers of resorption are elevated while formation markers remain low^53^. Interestingly, in treatment-naïve children with IBD, iliac crest biopsies revealed low % osteoclast and osteoid bone surfaces, suggesting low turnover^19^ therefore, potentially both the bone site and the current stage of the disease is important for capturing changes in bone turnover. At the distal femur metaphysis, pro-inflammatory cytokines were higher in DSS animals’ osteocytes vs. healthy controls, nearly 9-fold higher for TNF-α and 3-fold higher for IL-6. Additionally, osteoclastogenesis regulators RANKL and OPG were higher in osteocytes of DSS rats as well as sclerostin, an inhibitor of bone formation. These data concur with our previous findings in a different model (TNBS) that osteocyte proteins reflect a pro-inflammatory state during chronic gut inflammation and these changes in osteocyte proteins parallel the changes seen in osteoclast surfaces and bone formation rate^31,39^. Therefore, we hypothesize that gut inflammation, regardless of the cause, impacts bone potentially through osteocyte signaling within bone.

In our model, four weeks of DSS-induced IBD resulted in 9% lower ultimate load at the midshaft tibia and an even larger reduction in ultimate load (15%) at the femoral neck. To our knowledge, this is the first study to examine mechanical properties of bone in an animal model of IBD. Our data are consistent with the clinical literature demonstrating increased fracture incidence overall^22^, specifically at the hip^23^ and vertebral bone^24^. Linear regression models of our data demonstrate that body weight predicts 17% and 31% of the variability in tibial and femoral neck ultimate load, respectively. Additionally, the colon histopathology scores predicted 22% and 19% of the variability of tibial and femoral neck ultimate load, indicating that severity of the disease in the gut may influence the degree of change in bone mechanical properties. Similarly, in human patients, disease severity determined by the number of disease symptoms was associated with the risk of fracture^54^.

Previously, we identified the potential anti-inflammatory role exerted by exogenous irisin treatment in TNBS-induced IBD^39^. Since our TNBS model did not result in significant bone loss, we aimed to identify whether exogenous treatment with irisin could protect against bone loss in a more severe model of IBD. The irisin-treated DSS rats did exhibit significantly reduced colon histopathology scores compared to DSS alone, but those scores remained higher than those of healthy controls. Additionally, colonic TNF-α was mildly improved with modest restoration of lymphatic architecture. The irisin treatment did not protect against losses in body weight or vBMD in DSS-treated rats. Cancellous bone volume, while not different between DSS and DSS + Ir groups at the proximal tibia, was slightly higher at the 4^th^ lumbar vertebra (+22%) and femoral neck (+24%) compared to untreated DSS animals, although this did not reach statistical significance. Additionally, irisin-treated DSS rats did not exhibit improved bone mechanical properties compared to DSS alone. Interestingly, midshaft tibia cortical BMC and total area were lower in the irisin-treated control group versus untreated controls. Whether this difference is biologically significant will need to be addressed in future investigations.

While there was no impact of irisin treatment on mechanical properties or bone volume, irisin-treated DSS rats did have improved bone formation rate at the proximal tibia and femoral neck (113% and 115% higher, respectively, vs. untreated DSS animals), although BFR was still 42–57% lower than healthy controls. Osteoclast surfaces in DSS + Ir rats were not different from control levels, a 2-fold mitigation compared to the elevated osteoclast surfaces in the untreated DSS rats. Additionally, all osteocyte proteins in irisin-treated DSS rats were equivalent to those of the control rats; irisin treatment ameliorated the inflammatory status of bone due to DSS-induced colitis. This significant impact of irisin treatment on inflammatory status is also supported by linear regression analyses verifying that 63% of the variability in osteocyte TNF-α was predicted by colon histopathology scores. Higher colon histopathology scores with DSS-induced inflammation were matched by 13-fold higher osteocyte TNF-α while irisin-treated DSS rats had declines in both measures. Thus, our results demonstrate significant improvements due to exogenous irisin on measures of bone turnover and inflammatory proteins, but no preservation of bone mass. Given the slow turnover of bone tissue and the positive impact of exogenous irisin on bone formation rate and osteoclast surfaces, we hypothesize that a longer period of treatment with irisin may result in mitigation of loss of bone mass and mechanical properties.

The mechanisms of irisin’s influence on bone is just beginning to be elucidated, with some studies suggesting an anabolic effect of irisin on bone^40,43^ and protection of bone mass at doses higher than those used in this study^41^. The rats in our study received an irisin dose 4x greater than what we previously determined to be the circulating level in age-matched rats at rest. It is possible that a higher dose than this, as previous research has demonstrated^41^, would have increased/preserved bone mass; however, even our much lower dose was able to mitigate the inflammatory status of both gut and bone. More recent reports have also demonstrated αV integrins as irisin receptors in osteocytes and adipose tissues^42^. However, genetic deletion of FNDC5, the precursor for irisin, prevented ovariectomy-induced bone loss^42^ which highlights that much is still unknown about FNDC5/irisin and bone. Therefore, it is clear more research is needed to fully understand the role of irisin in bone and its potential therapeutic use.

Limitations of our study include the use of only one time point and only one irisin treatment dose. As addressed above, continuing the treatment for a longer period beyond 4 weeks may have allowed for changes in bone structure due to irisin treatment that we were unable to detect in our study. Additionally, earlier time points would be beneficial to determine the time course of cellular adaptations leading to the changes in osteoclast surfaces, bone formation rate, and osteocyte proteins we observed in our study after four weeks of DSS. Furthermore, future studies should also determine if there are sex-specific differences in bone outcomes during chronic gut inflammation. Lastly, higher doses of the administered irisin may have had stronger effects.

In conclusion, our data demonstrate that chronic DSS administration in rodents leads to the development of gut inflammation similar to ulcerative colitis, with elevated gut histopathology score, significantly lower body weight, mild colonic lymphatic and TNF-α inflammatory changes, reduced vBMD and cancellous bone volume, as well as reduced ultimate load. Additionally, high osteocyte pro-inflammatory markers, osteoclastogenesis regulators, and sclerostin were associated with low bone formation rate and high osteoclast surfaces in DSS rats. Treatment with exogenous irisin improved gut histopathology scores and mitigated podoplanin and TNF-α alterations in the colon. While ineffective in this model in protecting against loss of vBMD or mechanical properties, irisin treatment did significantly mitigate the inflammatory status of bone and improved bone turnover measures. These results demonstrate that severe, chronic gut inflammation results in impairments in bone mass and reduced strength in the growing skeleton. Furthermore, irisin represents a potential novel treatment for mitigating deleterious changes associated with chronic inflammatory conditions such as IBD.

## Materials and Methods

### Animals

Thirty-two Sprague-Dawley rats (male, 6 weeks old) from Envigo (Houston, Texas) were ordered and singly housed with 12-hour light-dark cycles. At 7 weeks of age, rodent diets were exchanged from the standard rodent chow of the facility (Teklad 2018; Envigo, Houston, TX, USA) to AIN93G chow (Research Diets, Inc., New Brunswick, NJ, USA). At 8 weeks of age, animals were grouped two-ways (n = 16/group): Control (Con) and IBD induced via 2% dextran sodium sulfate (w/v) in drinking water (DSS). All animals remained on their respective drinking water for 4 weeks. During the second week of DSS treatment, half of each Con and DSS group (Con + Ir, DSS + Ir; n = 8/group) were given bi-weekly intraperitoneal injections of recombinant irisin (Adipogen, San Diego, CA, USA) intraperitoneally as previously described^39^. Irisin doses were calculated off the baseline weight of rats to be 18 ng/mL and maintained throughout the course of the study. Recombinant irisin injections were continued for the remaining three weeks of treatment. Fluorochrome calcein labels (Sigma Aldrich, St Louis, MO, USA) were injected intraperitoneally eight and three days prior to termination to label mineralized surfaces on bone. Following four weeks of treatment, animals were euthanized and tissues were collected. All animal procedures were approved by the Texas A&M Institutional Animal Use and Care Committee and conform to the NIH Guide for the Care and Use of Laboratory Animals.

### Tissue processing and histological analysis

Standard histopathology was completed on the colon to assess structural damage and indications of inflammation. Whole length colons were removed, processed (~3 cm length was used), and were scored 0–4 (0 being no damage, 4 being severe damage) from H&E stained sections (oriented in a “swiss-roll”) based on: epithelial structure (1–2: loss of goblet cells and/or structural modifications, 3–4: damage to epithelium in terms of missing cells, gaps, complete erosion/loss or fibrosis), crypt structure (1: minimal gaps between crypts, basal 1/3 damaged, intact epithelium, 2: inflammatory infiltrate, basal 2/3 damaged, 3: loss of crypt, beginning of fibrosis, 4: complete loss of crypt and epithelium), cellularity (1: mucosal infiltrate, 2: mucosal + submucosal, 3: pre-granuloma, 4: granuloma or swelling into epithelial layer from crypt), and edema (separation of muscularis mucosa with epithelial layer, thickness of submucosa and muscularis externa) as previously described^39^. Overall scores were adjusted to account for area of tissue affected (as the whole ~3 cm length was assessed)^39^. Undamaged sites were accounted for in score calculation as “% area involved” of damage for each damage score^39,55,56^. Sites were assessed at both 20x and 40x magnification fields, and all scores were conducted blindly to treatment groups.

### Colonic immunofluorescence

Immunofluorescence was performed to examine and quantify lymphatic structures (via podoplanin) and pro-inflammatory responses (via TNF-α) in the colon. As previously described, tissues from the colon were collected, flushed and processed for paraffin or OCT embedding; some tissues were fixed in 4% PFA overnight and embedded in paraffin^39^. The paraffin sections (10-um) were deparaffinized, blocked for 30 m with 2.5% Goat Serum:PBS at room temperature, and incubated overnight at 4 °C with primary antibody combinations of anti-Podoplanin (Novus Biologicals, Littleton, CO) and anti-TNF-α (LifeSpan BioSciences, Inc., Seattle, WA)^39^. Sections were then incubated with corresponding secondary antibodies for Mouse IgG1 Alexa Fluor-488 and Rabbit IgG Alexa-Fluor 633 (Fisher Scientific, Hampton, NH) for 1 h at room temperature in the dark, and subsequently mounted in Prolong Gold Antifade with DAPI and imaged by confocal microscopy (Olympus Fluoview 300, Tokyo, Japan)^39^. Images (1024 × 1024) were acquired at 20x objective at 5 random fields with z-stack slices of 2 microns. As previously described, z-stacks were imported into ImageJ v.1.51 and quantified consistently across groups (Fluorescence Integrated Density [ID] = region of interest area x the mean fluorescence intensity)^39^. Regions of interest positive for protein markers were selected from each image and measured via ImageJ. Images were delineated into two regions of interest: (1) mucosa and (2) submucosa, via the location of the muscularis mucosa. For cell counting, each image had cells counted using ImageJ’s “Analyze Particles” function utilizing equivalent size (μm^2^) and circulatory ranges. Five images per animal were analyzed, averaged, and considered an n = 1.

### Peripheral quantitative computed tomography

To examine volumetric bone mineral density, pQCT scans were completed on the proximal tibia and proximal femur. Right tibiae and right proximal femurs were saved in phosphate buffered saline in −35 °C. Once thawed, *ex vivo* pQCT scans of the proximal tibia metaphysis, mid-shaft tibia, and right femoral neck were completed on a Stratec XCT Research-M device (Norland Corp., Fort Atkinson, WI, USA). Metaphyseal volumetric bone mineral density (BMD) was measured at the proximal tibia from 4 slices located at least 1 mm distal of the growth plate. Three contiguous slices were averaged to provide one value for each variable at the proximal tibia metaphysis. One mid-shaft tibia slice was taken at approximately 50% of the total bone length. Three scans of the femoral neck were averaged together for each variable. Scans were completed at 2.5 mm/sec scan speed, 100 μm voxel resolution, and 0.5 mm slice spacing Measures obtained from the *ex vivo* pQCT scans include total bone mineral content (BMC) and total, cortical, and cancellous volumetric bone mineral density (vBMD).

### Three-point bend mechanical testing

To assess the mechanical properties of cortical bone, a three-point bend test was conducted at the tibia midshaft. Excised right tibias were saved in PBS at −18 °C. Tibias were thawed just prior to testing and measured with digital calipers to determine the anterior-posterior and medial-lateral periosteal diameters at the mid-diaphysis. Each bone was positioned with the lateral side resting on two supports spaced 18 mm apart. An Instron 3345 machine with a 1000 N static load cell (Norwood, MA, USA; Bluehill v. 2.14.582) were used to apply a quasi-static load of 2.54 mm/minute to the medial surface of the diaphysis at 50% of the total bone length. Tibiae were tested to failure (fracture). Load-displacement data were analyzed using a custom Matlab script (v. 9.3.0.713579 (R2017b), The Mathworks, Inc.; Natick, MA, USA). Stiffness (*k*; N/mm) was determined by calculating the slope of the load-displacement curve in the linear elastic region. Ultimate load (*F*_*ult*_; N) was defined as the largest force achieved before fracture.

Cross-sectional moment of inertia (CSMI) was determined from *ex-vivo* pQCT scans of each tibia at 50% of bone length (the same location tested during three-point bending). The average rectangular CSMI at the tibia mid-shaft was taken as one-half of the polar area moment of inertia from the pQCT scan.

Intrinsic material properties were calculated according to Euler–Bernoulli beam theory. Estimated elastic modulus (EM) and ultimate stress (US) were determined using the equations below,

1 $$EM=\frac{k{L}^{3}}{48\ast CSMI}$$

2 $$US=\frac{{F}_{ult}L(\frac{{D}_{ML}}{2})}{4\ast CSMI}$$

where *k* is stiffness, *L* is the support span (18 mm), and *D*_*ML*_ is the measured medial-lateral periosteal diameter.

### Femoral neck mechanical testing

To further characterize bone mechanical properties, a compression test at the femoral neck was completed. Excised right proximal half femurs were saved in PBS at −18 °C. The diaphysis portion of each proximal femur was firmly inserted into a metal support which held the shaft of the bone upright. A compressive load was applied to the tip of the femoral head, parallel to the femoral shaft long axis, at a rate of 2.54 mm/min. The same Instron 3345 was used with a 100 N static load cell (Norwood, MA, USA; Bluehill v. 2.14.582). Only ultimate load (N) was reported from this test. Further measures were not calculated due to the complex combined loading conditions of shear, bending, and compression.

### Dynamic and static cancellous histomorphometry

Histomorphometry was completed at various bone sites to assess the impact of the DDS ulcerative colitis model on cancellous bone formation and resorption. For cancellous histomorphometry measures, undemineralized left proximal tibia, 4th lumbar vertebra, and left proximal femur were fixed in 4% phosphate-buffered formalin for 24 hours and then subjected to serial dehydration and embedded in methyl methacrylate (J.T. Baker, VWR, Radnor, PA, USA). Serial frontal sections were cut 8 μm-thick and left unstained for fluorochrome calcein label measurements. The histomorphometric analyses were performed using OsteoMeasure Analysis System, version 3.3 (OsteoMetrics, Inc., Atlanta, GA, USA). For the proximal tibia an area of approximately 8 mm^2^ at 20x magnification was analyzed while an area of approximately 3mm^2^ was used for both L4 and the femoral neck. At all sites, endocortical edges and primary spongiosa were not included in the region of interest. All histomorphometric data was collected and analyzed as previously described^31,39,57,58^. Total bone surface (BS), single-labeled surface (sLS/BS), double-labeled surface (dLS/BS), mineralized surface (MS/BS), and interlabel distances were measured at 20x magnification. Mineral apposition rate (MAR) was calculated from the interlabel distance and time of labels. Bone formation rate (BFR/BS) was determined by multiplying %MS/BS by MAR. Additionally, 4 μm-thick sections were treated with von Kossa stain and tetrachrome counterstain to measure cancellous bone volume (BV/TV) and osteoid (OS/BS) and osteoclast (Oc.S/BS) surfaces as a percent of total cancellous surface measured at 40x magnification. All analyses were completed by the same individual. All nomenclature for cancellous histomorphometry follows standard usage^59^.

### Dynamic cortical histomorphometry

Undemineralized left distal tibia were fixed in 4% phosphate-buffered formalin for 24 hours and then serially dehydrated and embedded in methyl methacrylate. Cross sections of the bone closest to the mid-shaft were made on an IsoMet Low Speed Saw (Buehler, Lake Bluff, IL, USA) approximately 100 µm thick. Cross sections were analyzed at 20x magnification using OsteoMeasure Analysis System, version 3.3 (OsteoMetrics, Inc., Atlanta, GA, USA) for MS/BS and MAR; BFR/BS was calculated as stipulated above.

### Bone immunohistochemistry

To assess the inflammatory status of bone, IHC of osteocytes was completed for assessment of pro-inflammatory cytokines (TNF-α and IL-6), osteoclastogenesis regulators (RANKL and OPG), a bone formation inhibitor (sclerostin), and a marker of apoptosis (annexin V). Left distal femora were fixed in 4% phosphate-buffered formalin for 24 hours at 4 °C and then decalcified in a sodium citrate/formic acid solution for approximately 14 days then stored in 70% ethanol. Sections were then further dehydrated in Thermo-Scientific STP 120 Spin Tissue Processor, paraffinized via a Thermo Shandon Histocenter 3 Embedding tool, sectioned to approximately 8 µm thickness, mounted on positively charged slides, and immunostained using an avidin-biotin method as previously described^31,39,57,58^. Briefly, samples were rehydrated, peroxidase inactivated (3% H_2_O_2_/Methanol), permeabilized (0.5% Triton-X 100 PBS), blocked with species-appropriate serum for 30 minutes at room temperature (Vectastain Elite ABC, Vector Laboratories, Burlingame, CA, USA) and incubated overnight at 4 C with primary antibodies: polyclonal rabbit anti-rat TNF-α, (LifeSpan BioSciences, Inc, Seattle, WA, USA), polyclonal rabbit anti-IL-6 (Abcam, Inc, Cambridge, MA, USA), rabbit polyclonal anti-RANKL (Abcam), rabbit polyclonal anti-OPG (Biorbyt, San Francisco, CA, USA), polyclonal goat anti-mouse sclerostin (R&D Systems, Minneapolis, MN, USA), and polyclonal rabbit anti-annexin V (Abcam). On the subsequent day, sections were incubated at room temperature for 45 minutes with the appropriate species’ biotinylated anti-IgG secondary antibody according to manufacturer specifications. Peroxidase development was performed with an enzyme substrate kit (DAB, Vector Laboratories). Counterstaining was conducted with 0.2% methyl green counterstain (Vector Laboratories) for 90 seconds; sections were subsequently dehydrated into organic phase and mounted with xylene-based mounting media (Polysciences, Warrington, PA, USA). Negative controls for all antibodies were completed by omitting the primary antibody. Sections were analyzed as the percentage of osteocytes stained positively for the protein within a 4 mm^2^ region of interest within the distal femur cancellous bone. All analyses were completed by the same individual.

### Statistical analyses

Data were analyzed as a 2 × 2 factorial design (DSS by irisin). If the model 2 × 2 ANOVA was statistically significant (p < 0.05), main effects for DSS, irisin, and DSS*Ir interaction were recorded as well as all pairwise comparisons. Regression analyses were completed on end point body weights and gut histopathology scores on tibia and femoral neck ultimate load. A new model was accepted when the adjusted R^2^ increased and all slopes in the model were significant (p < 0.05). If these criteria were not met, the previous model was accepted. A separate regression was completed with gut histopathology and TNF-α-positive osteocytes. Statistical analyses were completed on SPSS Statistics 25 (IBM; Armonk, NY, USA). All data are represented as mean ± standard deviation.

## Supplementary information

Supplementary Dataset 1
