# Supplementary material for: DSS-induced colitis produces inflammation-induced bone loss while irisin treatment mitigates the inflammatory state in both gut and bone
Source: Sci Rep. 2019 Oct 22;9:15144. doi: 10.1038/s41598-019-51550-w (PMC6805923; doi:10.1038/s41598-019-51550-w)
Supplement: Supplementary file 1 — Supplementary Dataset 1 [file 41598_2019_51550_MOESM1_ESM.docx]

**DSS-induced colitis produces inflammation-induced bone loss while irisin treatment mitigates the inflammatory state in both gut and bone**

Corinne E. Metzger*†^1^, S. Anand Narayanan,*†^2^, Jon P. Elizondo^3^, Anne Michal Carter^1^, David C. Zawieja^2^, Harry A. Hogan^3^, Susan A. Bloomfield^1^

†Authors contributed equally to this work

**Affiliations:**

^1^Department of Health and Kinesiology, Texas A&M University, College Station, TX, USA.

^2^Department of Medical Physiology, Texas A&M University - Health Science Center, Temple, TX, USA.

^3^Departments of Mechanical/Biomedical Engineering, Texas A&M University

**Supplemental Data**

Supplemental Table 1. Body weights measured in grams. *Indicates different from both Con and Con+Ir.

| **Group** | **Baseline** | **Week 1** | **Week 2** | **Week 3** | **Week 4** |
| --- | --- | --- | --- | --- | --- |
| *Con* | 212 ± 8 | 256 ± 15 | 303 ± 16 | 321 ± 20 | 343 ± 27 |
| *Con+Ir* | 218 ± 7 | 267 ± 14 | 303 ± 13 | 331 ± 17 | 352 ± 19 |
| *DSS* | 216 ± 9 | 263 ± 8 | 290 ± 14* | 285 ± 17* | 303 ± 22* |
| *DSS+Ir* | 215 ± 5 | 260 ± 5 | 282 ± 7* | 276 ± 25* | 290 ± 33* |

Supplemental Table 2. Average daily food intake over one week measured in grams. *Indicates different from Con and Con+Ir.

| **Group** | **Baseline** | **Week 1** | **Week 2** | **Week 3** | **Week 4** |
| --- | --- | --- | --- | --- | --- |
| *Con* | 18.2 ± 3.6 | 17.8 ± 2.3 | 18.6 ± 0.7 | 18.0 ± 1.9 | 16.7 ± 1.9 |
| *Con+Ir* | 19.5 ± 1.2 | 18.1 ± 1.2 | 18.2 ± 1.8 | 18.3 ± 1.7 | 17.6 ± 1.6 |
| *DSS* | 19.5 ± 1.2 | 17.2 ± 1.2 | 15.1 ± 2.2* | 16.1 ± 1.5 | 15.6 ± 2.8 |
| *DSS+Ir* | 19.1 ± 0.9 | 16.5 ± 0.8 | 13.4 ± 4.2* | 15.2 ± 3.2* | 13.9 ± 3.6 |


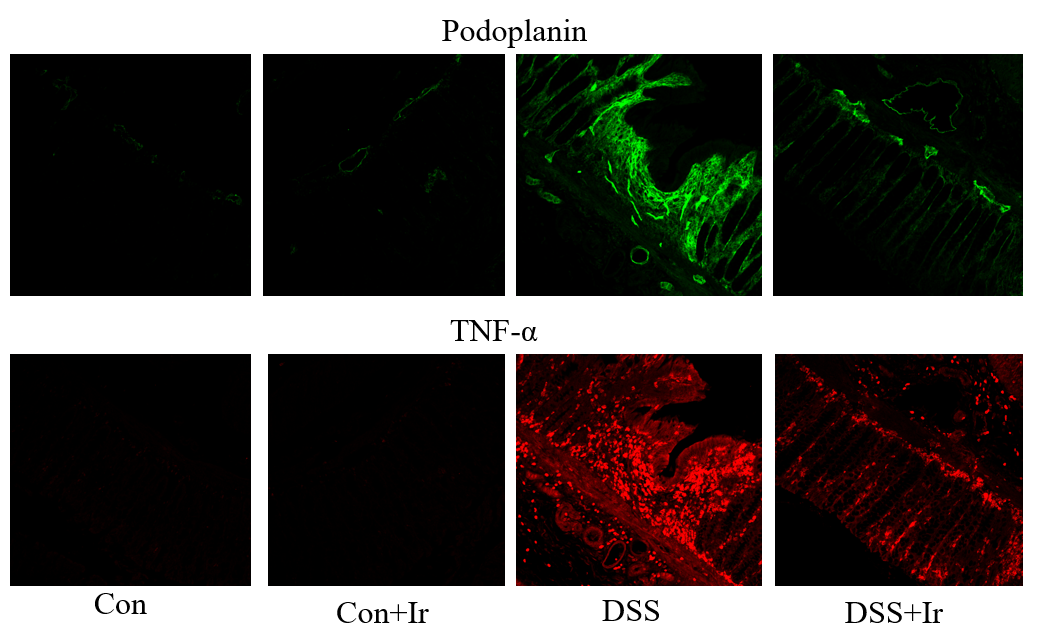


Representative images of podoplanin (top, green) and TNF-α (bottom, red) in the mucosa of the colon. For quantitative data, see Figure 2.
